# Anthelmintic Activity In Vivo of Epiisopiloturine against Juvenile and Adult Worms of *Schistosoma mansoni*

**DOI:** 10.1371/journal.pntd.0003656

**Published:** 2015-03-27

**Authors:** Maria A. Guimarães, Rosimeire N. de Oliveira, Leiz M. C. Véras, David F. Lima, Yuri D. M. Campelo, Stefano Augusto Campos, Selma A. S. Kuckelhaus, Pedro L. S. Pinto, Peter Eaton, Ana C. Mafud, Yvonne P. Mascarenhas, Silmara M. Allegretti, Josué de Moraes, Aleksandar Lolić, Tatjana Verbić, José Roberto S. A. Leite

**Affiliations:** 1 Biotechnology and Biodiversity Center Research, BIOTEC, Federal University of Piauí, Parnaíba, Piauí, Brazil; 2 Department of Animal Biology, Institute of Biology, State University of Campinas, Campinas, São Paulo, Brazil; 3 Graduate Program in Biotechnology, RENORBIO, Focal Point Federal University of Piauí, Teresina, Piauí, Brazil; 4 Collegiate Academic Medicine, Federal University of São Francisco Valley, Campus Paulo Afonso, Paulo Afonso, Bahia, Brazil; 5 Faculty of Medicine, University of Brasilia, UNB Campus Dacy Ribeiro, Brasília, Distrito Federal, Brazil; 6 Adolfo Lutz Institute, Central Laboratory, São Paulo, Brazil; 7 UCIBIO, REQUIMTE, Department of Chemistry and Biochemistry, Faculty of Science, University of Porto, Portugal; 8 Group of Crystallography, Institute of Physics of São Carlos, University of São Paulo, São Carlos, São Paulo, Brazil; 9 Research Center for Neglected Diseases (NPDN/FACIG), Guarulhos, São Paulo, Brazil; 10 Faculty of Chemistry, University of Belgrade, Belgrade, Serbia; McGill University, CANADA

## Abstract

Schistosomiasis is a serious disease currently estimated to affect more that 207 million people worldwide. Due to the intensive use of praziquantel, there is increasing concern about the development of drug-resistant strains. Therefore, it is necessary to search for and investigate new potential schistosomicidal compounds. This work reports the in vivo effect of the alkaloid epiisopiloturine (EPI) against adults and juvenile worms of *Schistosoma mansoni*. EPI was first purified its thermal behavior and theoretical solubility parameters charaterised. In the experiment, mice were treated with EPI over the 21 days post-infection with the doses of 40 and 200 mg/kg, and 45 days post-infection with single doses of 40, 100 and 300 mg/kg. The treatment with EPI at 40 mg/kg was more effective in adult worms when compared with doses of 100 and 300 mg/kg. The treatment with 40 mg/kg in adult worms reduced parasite burden significantly, lead to reduction in hepatosplenomegaly, reduced the egg burden in faeces, and decreased granuloma diameter. Scanning electron microscopy revealed morphological changes to the parasite tegument after treatment, including the loss of important features. Additionally, the in vivo treatment against juvenile with 40 mg/kg showed a reduction of the total worm burden of 50.2%. Histopathological studies were performed on liver, spleen, lung, kidney and brain and EPI was shown to have a DL50 of 8000 mg/kg. Therefore EPI shows potential to be used in schistosomiasis treatment. This is the first time that schistosomicidal in vivo activity of EPI has been reported.

## Author Summary

Schistosomiasis, a disease caused by worms from the genus *Schistosoma*, is one of the most widespread neglected tropical diseases in the world. Due to the intensive use of praziquantel, some reports of drug resistance have emerged. Thus it is important to search for new chemotherapeutic agents for the treatment disease. This research showed that epiisopiloturine, an imidazole alkaloid extracted from the leaves of *Pilocarpus microphylus*, has activity against *S*. *mansoni* in vivo. This material is produced as a by-product of an existing commercial process, and thus can be produced in large quantities at relatively low cost. The low toxicity and high drug tolerance in mammals shows the potential of epiisopiloturine as a new candidate schistosomicidal compound.

## Introduction

Schistosomiasis is a disease mainly found in tropical regions, whose infectious agents are *Schistosoma* spp., including *Schistosoma mansoni* [1]. This disease is currently one of the most widely occurring neglected tropical diseases with high levels of incidence in Asia, Africa and Latin America. Studies have shown that more than 207 million people have been infected worldwide and about 779 million people are liable to infection [2].

Currently praziquantel (PZQ) is effective against all specie of *Schistosoma*. Presenting good efficacy and low toxicity, it is currently the reference drug for schistosomiasis treatment [3–6]. However, as PZQ is the only drug used in therapy against schistosomiasis, concern about the development of drug resistance has been reported [7,8].

The search for new antischistosomal drugs has led to the study of natural substances such as artemisinin and its derivatives (e.g. artemether, artenusate), curcumin, phytol and epiisopiloturine (EPI) has been intensified [9–14]. We have been specifically interested in EPI which is an imidazole alkaloid extracted from *Pilocarpus microphyllus*, whose in vitro activity against *S*. *mansoni* at the concentration of 150 μg/mL led to the observation of dead parasites, and at the sub-lethal dose, prevented egg laying [14]. Moreover, this alkaloid showed potent anti-inflammatory activity [15], which might help combat the granuloma and inflammatory reaction caused by *S*. *mansoni* eggs.

Despite this promising evidence of in vitro activity, nothing has been reported until now about in vivo antischistosomal activity of EPI. In this paper, we describe the in vivo schistosomicidal activity of EPI against adult and juvenile *S*. *mansoni* worms for the first time. We also show scanning electron microscopy data revealing action of the alkaloid on the parasite tegument. Furthermore, the effects of the alkaloid in egg laying, reduction of hepatosplenomegaly, histopathology analysis and analysis of granulomas, in treated mice, are also characterised.

## Materials and Methods

### Purification and Characterization of EPI

This step was based on Véras et al., 2013 [16] by preparative high performance liquid chromatography—HPLC (SHIMADZU Prominence, SIL-10AF, CTO-20A, DGU-20A5, LC-6AD, CBM-20A, SPD-20A, Tokyo, Japan), and the molecular mass confirmation was performed by spectrometry (AmaZon SL, Bruker Daltonics, Bremen, Germany).

### Potentiometric pK_a_ determination

The acidity constant of EPI was potentiometrically determined in MeOH:H_2_O (1:1, *v*:*v*) at *t* = 25±1°C and at constant ionic strength (*I* = 0.1 M (NaCl)). Solutions of NaOH (0.1 M) and HCl (0.1 M) were prepared in MeOH:H_2_O (1:1, *v*:*v*) and potentiometrically standardized. EPI was dissolved in methanol and diluted with the equivalent volume of aqueous 0.2 M NaCl (*c*_EPI_ = 9.7789×10^-4^ M). Titrations were performed using CRISON pH-Burette 24 2S equipped with CRISON 50 29 micro-combined pH electrode (CRISON INSTRUMENTS, S.A. Spain). The electrode was calibrated by means of a strong acid—strong base titration, using GLEE—glass electrode evaluation software [17]; standard potential *E* ^0^ = 395.7 mV, slope factor of the electrode (actual slope divided by ideal Nernstian slope) *s*_f_ = 1.010, and p*K*_W_ 13.84±0.01 obtained as mean values of four titrations.

Prior to titration, 100.0 μL of the standard 0.1 M HCl solution was added to 4.00 mL of the EPI solution. All probes were titrated with 2.0 μL increments of the standard 0.1 M NaOH solution in 2.9–11.2 pH range. HyperQuad 2008 software [18] was used to calculate the value of acidity constant (p*K*_a_) from four repeated titrations.

A theoretical pKa study was performed. The ChemAxon method [19,20], implemented in the MarvinSketch package (Marvin 5.4.1.1, 2011, ChemAxon (http://www.chemaxon.com), was applied. This method is based on empirically calculated physico-chemical parameters (mainly partial charges) that are obtained from ionization site specific regression equations, it uses three types of calculated parameters (intramolecular interactions, partial charges and polarizabilities) to determine the micro ionization constants pKa of monoprotic molecules. In addition, we calculated the intrinsic solubility for the compound, which is a crucial solubility parameter. The prediction uses a fragment-based method that identifies different structural fragments in the molecule and calculates their solubility contribution. The implementation is based on the article of Hou et al., 2004 and it can found in ChemAxon package [21].

In addition, the thermal properties of EPI were evaluated. Differential scanning calorimetry (DSC) and thermal gravimetric analysis (TGA) were used to determine the thermal mass loss, as well as to study the EPI thermal decomposition to determine if it’s possible that polymorphic forms were present. Simultaneous DSC/TGA and DSC analysis were carried out with an initial sample mass of 5.0 mg in alumina pans (90 lL). A SDT-Q600 calorimeter, allowing simultaneous measurement of weight change and differential heat flow (TA Instruments) was applied in these studies. Experimental parameters for TGA curve included the mass used of 5 mg, heating rate of 10°C min^-1^ under N_2_ flow (50 mL min^-1^) and final temperature of 1000°C.

### Parasites, intermediate and definitive hosts

In this study the BH strain of *Schistosoma mansoni*, originated from Belo Horizonte, Minas Gerais, Brazil was used. The life cycle of *S*. *mansoni* was maintained in *Biomphalaria glabrata* snails at the Department of Animal Biology, IB, Unicamp. As the definitive host, Balb/C mice female, weighing ~ 20 g and 4 weeks of age, were previously infected exposure to a suspension containing approximately 70 cercaria using the tail immersion technique as described by Oliver and Stirewalt, 1952 [22]. The experiments were approved by the Ethics Commission for the Use of Animals (CEUA/UNICAMP, protocol no 2170–1), as they were in accordance with the ethical principles of animal experimentation adopted by CEUA.

### In vivo treatment with the EPI

#### Treatment of adult worms

For in vivo treatment of adult worms, the animals were treated 45 days post-infection and single doses of 40, 100 and 300 mg/kg of EPI were used. The dose of 40 mg/kg was selected based on previous studies describing the activity of PZQ in humans [23], and also because it is a commonly prescribed dosage for individuals infected with the disease. For both treatments, the samples was solubilized in solution of phosphate buffered saline (PBS) with 0.1% Tween-80 Polysorbate (Merck^®^). Forty-five days post infection the animals were divided into 4 main groups, with 10 animals each: infected untreated mice served as controls (Group I) and test subjects were EPI-treated mice treated with single doses of 40, 100 and 300 mg/kg (Group II, III and IV, respectively). All animals groups were treated orally. The group I consisted of a control group only received PBS. It is important to mention that in the experiment with EPI 100 mg/kg three mice died.

#### Treatment of juvenile worms

For in vivo treatment against juvenile worms, single doses of 40 and 300 mg/kg of EPI were used. Twenty-one days post infection the animals were divided into 3 main groups, with 8 animals each: infected untreated mice served as controls (Group I) and EPI-treated mice with 40 and 300 mg/kg (Group II and III). Group I consisted of a group only treated with PBS. The mice were treated orally in the same manner described in item a.

### Worm and egg burden estimation

The animals of all groups were sacrificed 15 days post-treatment, the mice were euthanized using cervical dislocation and *S*. *mansoni* worms were retrieved by perfusion of the hepatic portal system and mesenteric veins according to Pellegrino and Siqueira, 1956 [24]. The percentage of worm reduction (WR) was calculated according to Delgado et al., 1992 [25]. The counting of the eggs eliminated at the faeces was performed on the day of the analysis of the treatments. The faeces were collected before the euthanasia and were examined, utilizing the Kato-Katz quantitative method [26]. The slides were examined for *S*. *mansoni* eggs under a light microscope. After the perfusion portal system, a fragment of the intestine (2 cm) was cut off and processed for oogram. Eggs were then counted and classified according to different stages of development [27].

### Scanning Electron Microscopy (SEM)

Parasites were initially fixed in AFA solution, a 2:9:30:59 mixture of acetic acid, formaldehyde, ethanol (95%) and distilled water [28]. AFA was removed and replaced with a secondary fixative, osmium tetroxide (OsO_4_) in cacodylate buffer 100mM, pH 7.3. Secondary fixing took place for 2 h with gentle shaking. OsO_4_ was removed and replaced with cacodylate as above—twice. The cacodylate buffer was then replaced with ultrapure water—twice. Samples were then air-dried from water onto adhesive carbon tape, coated with gold-palladium for conductivity, and imaged in the SEM. The SEM was a FEI quanta 400 SEM, operating in secondary electron mode. Samples were imaged with a working distance of 10–15 mm and an accelerating voltage of 15kV. The back-scattered electron detector was removed from the SEM to increase the field of view.

### Hepatic histopathology and granuloma measurement

Regarding the histological analysis, liver samples (left lobe) were taken from of three mice: treated and control group (infection untreated). The samples were processed for histopathology following standard techniques [29], stained with hematoxylin and eosin and analysed with the Leica^®^ DM500-ICC50 HD photomicroscope. Measurements were made only for granulomas containing a single egg in their centers. The mean diameter (μm) of each granuloma was obtained by measuring two diameters of the lesions at right angles to each other with the help of an ocular micrometer [30].

The percentage of degenerated eggs was calculated from the number of degenerated miracidia (acellular or partially or completely degenerated) using the formulation: mean number of degenerated ova/mean number of granulomas X 100 [31].

### Toxicity and histopathological studies

For the determination of lethal dose capable of killing 50% of animals (DL_50_), Swiss female mice (n = 4), aged 3.5 ± 0.5 months and weighing 29.8 ± 1.3 g were used, based on Valadares (2006) [32]. Concentrations higher than the therapeutic dose of 40 mg/kg, which was previously identified in treating animals infected with *S*. *mansoni* were used in the assay. Initially, EPI was diluted in 10% DMSO and subsequently in 0.9% saline solution; the concentrations (0, 70, 130, 270, 530, 1070, 2130, 4270, 8000 mg/kg) were calculated based on the average weight of the animals and administered to animals intraperitoneally. During the assay, the animals were monitored periodically to identify visible clinical signs in the heart (abnormal heartbeat), lung (abnormal respiratory rate) and motor system (ataxia and/or prostration). Histopathological studies were performed on liver, spleen, lung, kidney and brain. Some animals were killed due to the administration of higher concentration, while the rest of them were killed 7 days after the intraperitoneal injection of the drug. Hematoxilin & Eosin staining was used with 100X magnification. [33].

### Statistical analysis

Statistical tests were performed with Graphpad Prism (version 5.0) software. Dunnet’s test was used to analyze the statistical significance of differences between mean experimental and control values and by applying Tukey’s test for multiple comparisons with the level of significance set at P < 0.05.

## Results

### Characterization of EPI

The HPLC retention time and characteristic fragments generated through mass spectrometry is in accordance with previously described data [16, 34].

**Fig 1:**
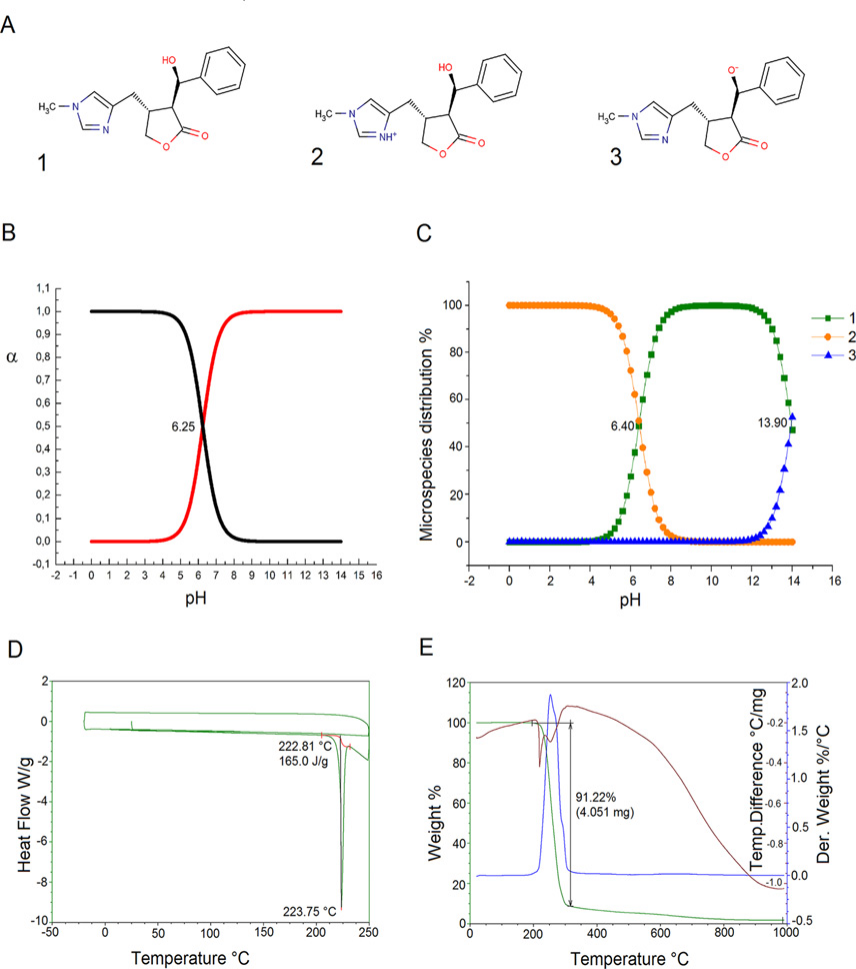
Neutral and ionic forms of EPI (A). Potentiometric pKa determination (B). Illustration of the pKa values of EPI predicted using the ChemAxon method (C). This graph shows the variation in the of distribution species vs pH values, and indicates that the ChemAxon predicted pKa values are respectively pKa1 = 6.40 and pKa2 = 13.90 (C). DSC (D) and DSC-TGA (E) data illustrating the thermal degradation that occurs around 220°C in a single step.

In the physiological pH range, EPI acts as a base with an sp^2^ nitrogen in the imidazole ring that can be protonated (Fig. 1A). According to potentiometrically determined p*K*_a_ value (p*K*_a_ 6.25 ± 0.05) and the distribution diagram shown on Fig. 1B, at pH 7.4, 93.5% of EPI is present in its molecular form and 6.5% in the protonated form.

The pKa calculation for EPI presents 3 different ionic forms shown in Fig. 1B. By plotting the theoretical titration curves, i.e. the ratio of the ionized and neutral forms versus the pH (Fig. 1C), one can identify the ionic species which are present in the mixture at each pH value. From this plot we identify the predicted pKa values as pKa1 = 6.4 and pKa2 = 13.9. The microspecies distribution at pH = 7.40 was 1 = 90.55%, 2 = 9.45%and 3 = 0.0%. The solubility was also calculated and the results show that EPI has a high apparent solubility in water at pH 7.4 (higher than 0.06 mg/mL), with intrinsic solubility equal to -1.76 logS units. An analysis of the thermal data indicates that EPI degradation occurs in one step at a high temperature (Fig. 1D). The single stage involves a mass decay of 91.22% (calculated: 4.051 mg; onset: 222.81°C) associated with an endothermic DTA signal (Fig. 1E). The analysis of this thermal data indicates that EPI can be used safely, with no risk of thermally-activated degradation or alteration in the organism.

### Effect of EPI on juvenile *S*. *mansoni*

The mice treated with the dose of 40 and 300 mg/kg of EPI, after 21 days of infection showed significant differences (P<0.01 and P< 0.01) regarding the reduction of total worm burden. The treatments performed against juvenile worms showed a moderate reduction of the total worm burden of 50.2% (SD: 14.9) and 46.3% (SD: 16.2) when the mice received EPI at 40 and 300 mg/kg, respectively, compared to the untreated infected control group. In Table 1, all results are summarized.

**Table 1:** Effects of EPI against juvenile *S*. *mansoni* BH strain (treated 21 days post infection).

Sample	Dose	Mean worm burden ± SEM (liver and porto-mesenteric)				Reduction in total worm burden
		Male	Female	Couples	Total Worms	
Control	0 (PBS only)	10.2±2.8	3.9±0.6	9.6±1.8	32.9±3.7	-
EPI	40 mg/kg	2.2±0.5*	2.4±0.6	5.9±0.7	16.4±1.7**	50.2%
EPI	300 mg/kg	6.8±1.4	0.9±0.3***	5.0±1.0*	17.6±1.4**	46.3%

Values are presented as mean ± SEM. Number of animals/group = 8.

Asterisks indicate that difference compared to the control was significant with ***P<0.001, **P<0.01 and *P<0.05.

### Effect of EPI on adult *S*. *mansoni*

The efficacy of EPI was tested against the adult parasite life stage in an experimental mammalian host. After 45 days of infection, male and female worms had matured and paired, and eggs were found in the liver, intestine, and faeces. In mice infected by *S*. *mansoni*, there was a reduction in worm burden upon oral treatment by EPI, as shown in Table 2. After 45 days of infection, the treatments performed with EPI in doses of 40, 100 and 300 mg/kg reduced the worm burden of the mice to 70.0% (P<0.001) (SD: 15.7), 39.0% (P<0.01) (SD: 14.1) and 46.7% (P<0.001) (SD: 9.2) respectively.

**Table 2:** Effects of EPI against adult *S*. *mansoni* BH strain (45 days post infection).

Sample	Dose	Mean worm burden ± SEM (liver and porto-mesenteric)				Reduction in total worm burden
		Male	Female	Couples	Total Worms	
Control	0 (PBS only)	12.9±1.1	9.9±1.2	13.7±0.9	50.2±2.4	-
EPI	40 mg/kg	2.6±0.6***	2.2±0.7***	5.3±0.9***	15.2±2.5***	70.0%
EPI	100 mg/kg	7.1±1.0**	6.8±1.3*	8.7±0.7**	31.4±2.7***	39.0%
EPI	300 mg/kg	12.1±1.1	3.0±0.3***	6.1±08***	27.4±1.7***	46.7%

Values are presented as mean± SEM. Number of animals/group = 10. Group treated with 100 mg /kg 3 animals died.

Asterisks indicate that difference compared to the control was significant with *** P<0.001, **P<0.01 and *P<0.05.

### Effect of EPI on egg elimination and liver and spleen weight

The effect of EPI on egg development stages (oogram pattern) and fecal egg burden at 45 days post infection is shown in Table 3. In relation to the number of eggs excreted in the faeces, the results showed reductions of 80% (SD: 9.1), 45.7% (SD: 22) and 59.6% (SD: 35.2) in response to treatments with 40, 100 and 300 mg/kg of EPI, respectively. It can be seen that in the groups treated by EPI at 40, 100 and 300 mg/kg, the percentage of immature eggs was significantly reduced to 24% (SD: 13.6), 16.6% (SD: 11.1) and 56.8% (SD: 13.7) of the total number of eggs, compared with infected untreated controls, in which 79% (SD: 6.1) of the eggs were immature. The oogram also showed significant increase in percentage of dead eggs in the mice treated with EPI (36% (SD: 9.9), 24% (SD: 5.6) and 12% (SD: 6.1) respectively) compared with the control group (2%) (SD: 1.2).

**Table 3:** Effect of treatment with EPI on oviposition by adults, 15 days post-treatment (45 days post infection).

Samples	Dose (mg/kg)	Reduction in number of eggs in faeces	Percentage of eggs developmental stage		
			Immature	Mature	Dead
Control	PBS	-	79.0%	19.0%	2.0%
EPI	40 mg/kg	80.0%***	24.0%***	40.0%	36.0%***
EPI	100 mg/kg	45.7%	16.6%***	59.4%	24.0%***
EPI	300 mg/kg	59.6%***	56.8***	31.2%	12.0%***

Number of animals/group = 10. Group treated with 100 mg /kg 3 animals died.

Asterisks indicate that difference compared to the control was significant with *** P<0.001

In addition, treatment with EPI 40 mg/kg decreases liver and spleen pathology. A significant reduction in the weight of liver (P<0.01) and spleen (P<0.01) of the group treated with EPI compared to the infected untreated control was demonstrated (Table 3). Treatment with EPI 100 mg/kg was significantly reduced only in the liver (P<0.05). On the other hand, mice treated with EPI 300 mg/kg did not see any reduction in the weight of these organs (Table 4). In short, EPI significantly decreases *S*. *mansoni* egg and worm burdens, as well as hepatosplenomegaly.

**Table 4:** Effect of treatment with EPI on relative organ weight.

Sample	Relative liver weight	Relative spleen weight
**Control**	1.88 ± 0.098	0.61 ± 0.034
**EPI 40 mg/kg**	1.60 ± 0.03**	0.45 ± 0.03**
**EPI 100 mg/kg**	2.00 ± 0.06*	0.61 ± 0.01
**EPI300 mg/kg**	1.78 ± 0.05	0.54±0.02

Asterisks indicate that difference compared to the control was significant with **P<0.01 and *P<0.05.

### Effect of EPI on the tegumental changes

**Fig 2:**
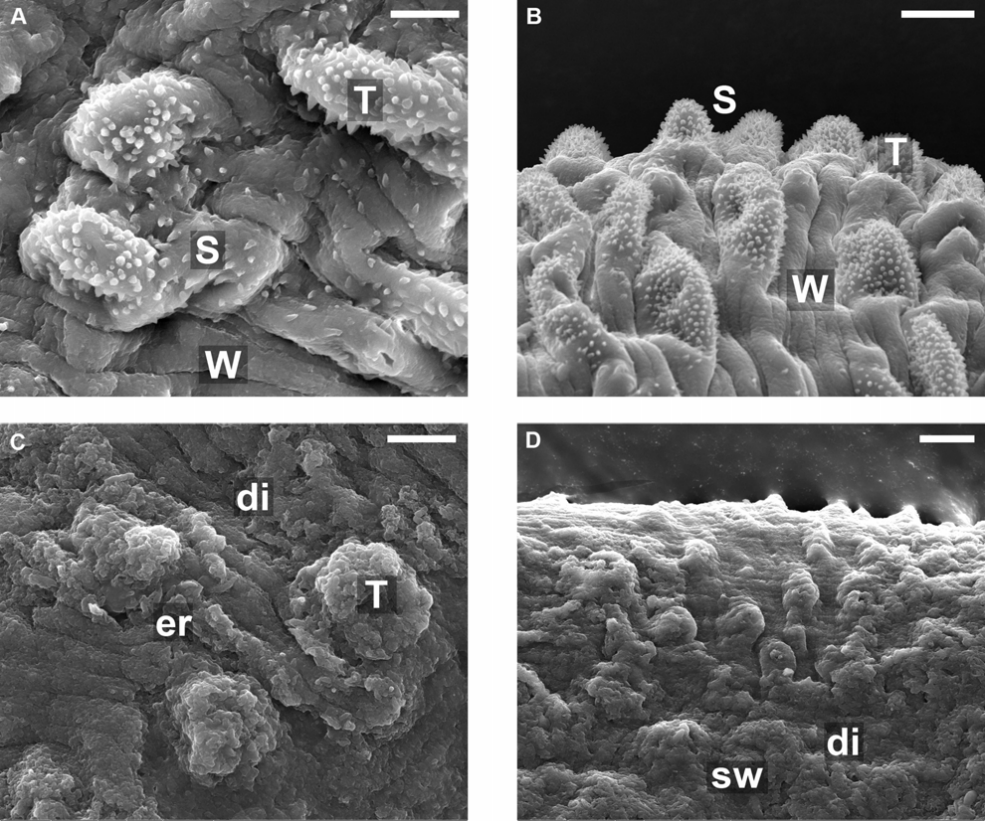
Scanning electron microscopy of the mid-body dorsal region of male *S*. *mansoni* worms recovered from a mouse 45 days post-infection by portal perfusion. A and B: Untreated (control) worms. C and D: worms from rodents treated with 40 mg/kg EPI. Scales bars in A and C show 5 μm, and in B and D show 10 μm. (A): dorsal tegumental surface showing tubercles (T), apical spines (s) and parallel wrinkles (w). (B) Lower magnification of (A) showing the same features regularly appearing across surface. (C) and (D): SEM of male worm after treatment with EPI: Tubercles (T) and the lateral tegument disintegrate (di) resulting in disappearance of the knobs, spines and many of the parallel-arranged wrinkles. Dorsal tegumental surface shows swelling (sw), and erosion (er) of the surface with the exposure of subtegumental tissue. Overall appearance quite different to that of the control worms, due to erosion of tubercules and loss of all apical spines.

The alterations of the tegument on the *S*. *mansoni* surface treated with EPI were analyzed by scanning electron microscopy (SEM). In control (untreated) parasites the tubercles and spines appeared intact, and at high magnification, the surface showed spiny tubercles and ridges between the tubercles which have a pitted appearance and bear sensory papillae (Fig. 2A and B). The tegument was dramatically damaged following the treatment with EPI, spines completely absent and tubercules badly damaged (Fig. 2C and D). The changes included the swelling of tubercles, and the loss of spines on tubercles, and sloughing and erosion. All worms examined showed shrinking with furrowing, extensive sloughing around the tubercles on the tegument exposing subtegumental tissues (Fig. 2C). In comparison with untreated worms, the overall structure of the worms treated by EPI showed far greater changes, with tegumental ridges no longer visible at low resolution (Fig. 2D), and far fewer intact tubercles visible, and no spines seen at all. It is also worth noting that control worms and EPI—treated worms were obtained whole.

### Hepatic histopathology and granuloma measurement

**Fig 3:**
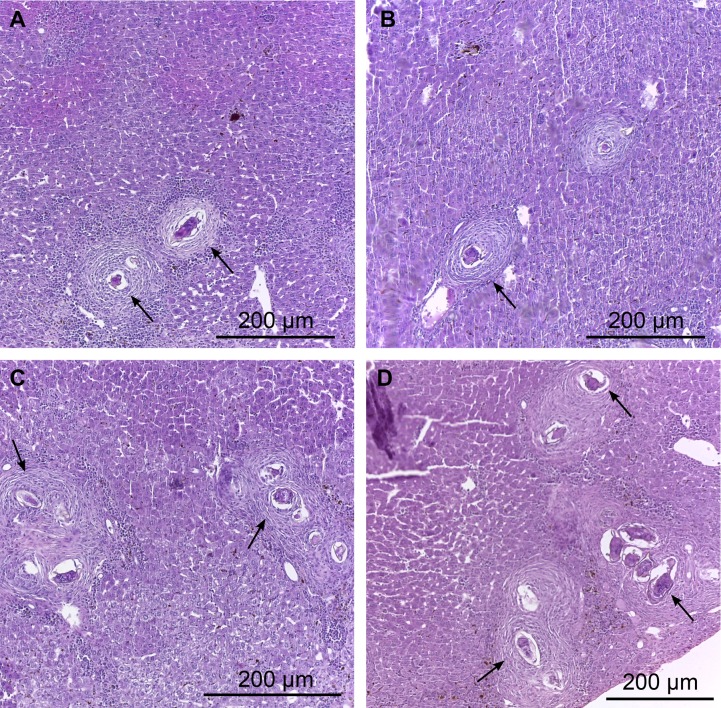
Photomicrographs of hepatic granulomas of mice liver 45 days post infection. A and B: Liver of treated infected animals (40 mg/kg). Note the necrotic-exudative phase with loose inflammatory infiltrate (macrophages, neutrophils and eosinophils) and areas with necrotic cells around the egg. C and D: Liver of untreated infected animals, the granulomas were found in the periportal area. Different stages of evolution of the granuloma, including granulomatous pre-exudative, necrotic-exudative, productive and healing by fibrosis were observed. Arrows indicate the presence of inflammatory cells.

Whereas the dose of 40 mg / kg led to a greater reduction in parasite burden, we evaluated the presence of granulomas in the liver of mice treated with this dose. A detailed study of histological sections of liver from the same area from BALB/c mice infected with *S*. *mansoni* and treated with dose of 40 mg/kg of EPI (Fig. 3A and B) and the untreated control (Fig. 3C and D) allowed the identification of granulomas at various stages of evolution containing infiltrate inflammatory compound of eosinophils, macrophages, neutrophils around the egg and more externally lymphocytes and fibroblasts. Based on the classification of Lenzi et al., 1998 [35], we identified in the liver histological sections granulomas containing some disorganized inflammatory infiltrate around the egg (pre-granulomatous phase initial reaction), excess diffuse inflammatory infiltrate (pre-granulomatous phase exudate), inflammatory infiltrating loose and necrotic cells around the egg (necrotic-exudative phase), initial deposition of collagen fibers by fibroblasts lining the inflammatory cells (productive phase) and thick bands of collagen fibers surrounding the eggs and their remains (phase healing by fibrosis).

Decreases in the granuloma sizes were observed in the livers of the group treated with EPI. Upon analysis of the images, it could be seen that the average diameter of the hepatic granulomas was significantly smaller (P<0.01) in the group treated with EPI, in comparison to the untreated infected control group (Table 5). Furthermore, most of the *S*. *mansoni* eggs from the animals treated with EPI showed of severe degeneration (71% versus 50% for the controls).

**Table 5:** Granuloma diameter (μm) in the liver of the mice from treated and untreated adult worm-infected groups 45 day post infection.

Sample	Granuloma diameter average (μm) ± SEM	Number of granulomas
Control infected Untreated	243.5 ± 3.23	68
EPI 40 mg/kg	194.0 ± 6.95**	30

Difference was significant ** *P*<0.01.

### Toxicity and histopathological studies

Toxicity and histopathology studies were carried out to assess the toxicological profile of EPI in vivo. The concentrations tested were considerably higher than the therapeutic doses used to measure effects on *S*. *mansoni*, since the toxicity of PZQ is low, with a DL_50_ around 2,400 mg/kg [36]. Intraperitoneal injection of EPI at a concentration equal to or higher than 530 mg/kg caused visible clinical changes such as tachycardia, tachypnea, ataxia and prostration. After a period ranging from 15 minutes to 6 hours, these changes were spontaneously reversed, eventually becoming imperceptible to observation, in a dose dependent way. The maximum concentration of 8,000 mg/kg killed 50% of animals with 18 hours of intraperitoneal injection of the drug.

**Fig 4:**
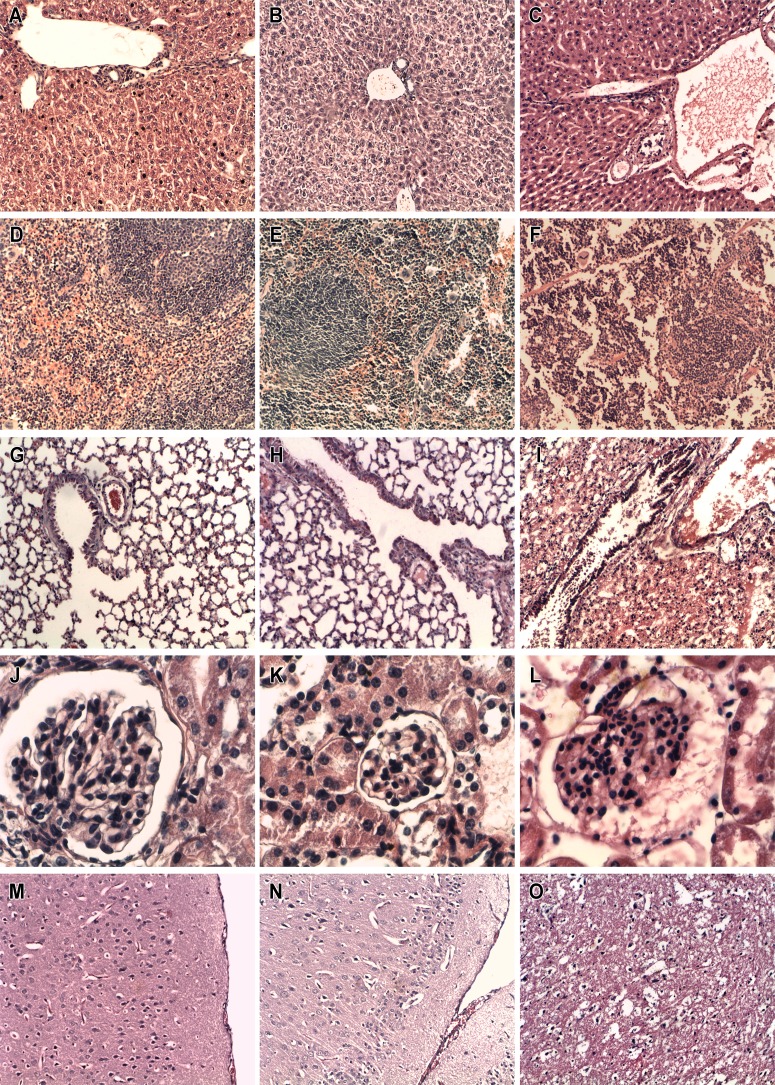
Histopathological study of organs sections of different groups of mice. Comparison between the histology of: liver (A,B,C), spleen (D,E,F), lung (G,H,I), kidney (J,K,L), and brain (M,N,O) obtained from Swiss mice (n = 4) treated with 0.0 (first column), 530 (second column) or 8000 (third column) mg/kg of EPI. The first and the second column refer to mice killed 7 days after assay and the third one represent organs from animals that died due to the administration of higher concentration of the drug. Light microscopy evaluation of these organs showed severe morphological changes in the spleen, lung, kidney and brain, but not in the parenchyma of liver in the animals treated with 8000 mg/kg of EPI. Focal alterations in the red pulp were observed in the spleen of animals treated with 530 mg/kg of the drug. Control group presented no morphological changes in the organs.

Fig. 4 shows images illustrating the histology of the liver (Fig. 4A, B and C), spleen (Fig. 4D, E, F), lung (Fig. 4G, H, I), kidney (Fig. 4J, K, L), and brain (Fig. 4M, N, O) treated with 0.0 (first column), 530 (second column) or 8000 (third column) mg/kg of EPI. The histopathological analysis showed that the animals treated with the highest concentration (8,000 mg/kg) showed generalized congestion and edema in the histological sections of lung, spleen, kidney, and brain; depression in the red pulp and uncountable cells undergoing apoptosis in the spleen, as well damage in the endothelium of the vessels in the liver were also observed (Fig. 4F). Other effects observed following 8,000 mg/kg injection included intra-alveolar hemorrhage and fibrin deposition in the lung (Fig. 4I) and focal hydropic degeneration of the kidney (Fig. 4L); no morphological changes were observed in the parenchyma of the liver (Fig. 4C). Animals treated with 530 mg/kg EPI showed focal depression of the red pulp (Fig. 4E) after seven days of intraperitoneal injection of the drug, while the other organs presented no morphological changes (Fig. 4B, H, K, N). The control group showed normal morphology in all organs analyzed (Fig. 4A, D, G, J, M).

## Discussion

The widespread use of PZQ for treatment and control schistosomiasis may select drug-resistant parasites [37]. In countries like Egypt, an increasing number of patients have displayed resistance to treatment with PZQ, apparently due in some cases to resistance inherent in particular strains, and in other cases to an immune response from the infected host, which reduces chemotherapy effectiveness [38]. Therefore, the discovery of new antiparasitic agents to treat schistosomiasis is an important avenue of research.

Plant compounds have attracted the attention of many researchers over the years for the development of new drugs, due of the variety of their chemical structures and their broad range of biological activities. Discovery of untapped natural sources that can be used in treatment of schistosomiasis remains a major challenge in the era of combinatorial chemistry and genomics [28].

We have evaluated the activity of a single dose of EPI against the laboratory model *S*. *mansoni* in vivo, firstly to determine the acute toxicity (DL_50_) and also to determine the effect of different concentrations of EPI (40, 100 and 300 mg/kg) on *S*. *mansoni* infected-mice.

Our results for the acute toxicity showed that intraperitoneal injection of EPI at a concentration equal to or higher than 530 mg/kg caused visible clinical changes such as tachycardia, tachypnea, ataxia and prostration, which were spontaneously reversed after 6 hours.

The treatment of *S*. *mansoni* infected-mice caused significant reductions in worm load, faeces egg load, and the frequency of egg developmental stages and reductions in hepatosplenomegaly. The effect on worm burden with single doses of 40 and 100 mg/kg of EPI showed there was no significant difference in susceptibility between males and females. However treatment with 300 mg/kg of EPI showed greater sensitivity by the female worms. Some susceptibility differences related to dose are described in the literature. For example, some studies [38–41] described that subcurative doses of PZQ act in the same proportion on males and females, with no significant differences in the therapeutic sensitivity between the genders. But Delgado et al. [25] also observed that the in vivo concentration of 250 mg/kg of PZQ acts preferentially on females of this parasite. Other works with antischistosomal drugs have also shown female worms with greater susceptibility than male worms [28, 31, 42, 43].

The in vivo treatments against immature worms of *S*.*mansoni* modify the proportion between male and female parasites. The treatments with the 40 and 300 mg/kg doses showed a moderate worm burden reduction, which did not significantly increase at the higher concentrations. (50.2% with SD: 14.9 and 46.3% with SD: 16.2, respectively). Utzinger et al. [44] observed that juvenile worms were not susceptible to PZQ. Probably the effect of EPI against juvenile worms can be an advantage in relation to PZQ.

At the end of the treatment, the weight of the liver and spleen of infected mice treated with 40 mg/kg of EPI were lower than of those from control infected mice. The anti-inflammatory properties of EPI may have helped in this effect. But, this reduction did not happen at doses of 100 and 300 mg/kg. Given the fact that *S*. *mansoni* leads to severe granulomatous inflammatory reactions in the liver, a compound with anti-inflammatory properties might be of great interest in the enhancement of the anti-inflammatory barrier. The immediate consequence was a reduction of the number and the size of granulomas in the liver and then a reduction of hepatosplenomegaly. Treatment with doses of 100 and 300 mg/kg did not show the same result compared with 40 mg/kg. This could indicate that the greater doses overcome the anti-inflammatory effect seen at lower doses, possibly due to a change in the partition of the drug when doses are increased [45].

Interestingly, looking at the total worm burden data (Table 1), greater decreases in worm burden were obtained at 40 mg/kg compared with either 100 or 300 mg/kg. The differences in the worm burdens between the two upper concentrations were not significant, although the decrease in both cases, although less than that seen with a dose of 40 mg/kg, was significant compared to the control. In addition, considering Table 2, we see that a similar dose dependence can be observed in the effect on egg laying. 100 and 300 mg/kg doses both lead to significant reductions in oviposition, but a greater effect was observed following the smaller dose of 40 mg/kg. Although inverse dose-response relationships are not unknown, they are unusual. In this case, the reason for this could be related to the administration method. The higher doses could lead to precipitation in the gut, leading to a failure to absorb. A very important future goal is to determine the pharmacokinetics of this drug in the model, to help explain these data. Further future experiments could also probe even smaller doses.

Although we were unable to observe significant toxicity at these treatment conditions, 3 animals did die after treatment with 100 mg/kg. It seems from this work that treatment with EPI at concentrations above 40 mg/kg do not offer any advantages in this model.

We carried out a comparison of the numbers of immature, mature and dead eggs present in the intestinal fragments of hosts—treated (45 days post-infection) and untreated. Our results demonstrated a significant decrease in the percentage of immature eggs in the intestinal tissue of the treated animals at all doses when compared to the untreated infected control groups. The decrease in the percentage of immature eggs in treated mice probably results from a decrease of worm burden and an effect on the reproductive fitness of *S*. *mansoni*. Probably the increase in the mature eggs compared to immature ones, happened as result of anterior oviposition. The results presented here concerning the reduction of eggs match those observed in other studies of effects of different compounds on *S*. *mansoni* [13, 46]. The reduction in oviposition is rather important for therapeutic use since the egg is responsible for transmitting the parasite and maintenance of its biological cycle. Furthermore, pathology associated with human schistosomiasis is thought to be due to the large number of eggs that become trapped in host tissues [47].

In addition, we evaluated the effect of EPI on egg load. The alkaloid significantly reduced (*P*<0.001) the number of eggs per gram of faeces compared to control. This remarkable reduction of fecal egg count could be related to decrease parasite load. Similar results were obtained by Moraes et al., 2014 [13] in studies with phytol.

Since the dose of 40 mg/kg led to a greater reduction in parasite burden, we evaluated the presence of granulomas in the liver of mice treated with this dose. The livers of EPI treated mice showed reduced granuloma diameters and a preserved typical lobular architecture. Eosinophils cells are known to play a major role in ovum destruction both in vitro [48] and in vivo [49] and amelioration of hepatic pathology and the presence of small granulomas with predominant eosinophils as a result of accelerated egg destruction upon repeated administration of soluble antigens of the parasite eggs (SEA) have been reported [50]. Therefore, there is the possibility that the increase in the number of degenerated eggs, as well as the decrease in the diameter of granulomas after treatment with EPI has occurred due to immunological interventions and immunomodulating actions. A similar effect was shown by Miranda et al., 2013 [51] in studies with the alkaloidic extract of *Solanum lycocarpum* fruits. This extract showed immunomodulatory effect on mice infected with *S*. *mansoni*.

The tegument is a prime target for drug studies, alterations in the surface topography of schistosome worms has been studied by several investigators [52–54]. The EPI doses tested exhibited a severe effect on the tegument of adult *S*. *mansoni* comprising of swelling of the tubercles and ridges, followed by loss of spines. Subsequently, the tubercles and ridges were broken and began to erode, leading to the formation of lesions. Eventually this resulted in sloughing of the tegument over large areas of the body surface. We speculate that the morphological changes found in the tegument can be a mechanism by which EPI leads to death of the worms. The damage to the tegument along the worm's body could have destroyed the defense of the worms, enabling subsequent attack by the immune system. The mechanisms involved in schistosomes death may be dependent on host antibodies or direct effect, [13]. Based on the premise that thioredoxin glutathione reductase (TGR) is an enzyme essential for the survival of the parasite *S*. *mansoni* [55], we did docking EPI with the TGR, but the EPI did not bind the enzyme and thus, we suppose that this is not the target. Thus, further studies are needed to elucidate the multiple mechanisms of action of EPI. This will be the subject of future study.

Toxicological evaluation of new drugs is one of the decisive steps of drug development. Filho et al., 2002 [56] evaluated three oxamniquine derivatives as novel schistosomicide agents when administered to mice. Oxamniquine has a DL_50_ value of 1,300 mg/kg, and the three new derivative compounds had even higher toxicities, with values of DL_50_ less than 1,200 mg/kg. PZQ, when administered to mice, has a DL_50_ around 2,400 mg/kg [35]. In our case, EPI was shown to have a far greater DL_50_ (8,000 mg/kg) and the mice were seen to have a high resistance to the toxic effects of EPI. The most dramatic histopathological alterations caused by EPI, such as generalized congestion and edema in the histological sections of lung, spleen, kidney, and brain, depression in the red pulp and the uncountable cells undergoing apoptosis in the spleen, intra-alveolar hemorrhage and fibrin deposition in the lung, focal hydropic degeneration of the kidney and damage in the endothelium of the vessels in the liver may be responsible for the toxicity at the highest concentration of 8,000 mg/kg. Below 530 mg/kg EPI caused no toxicity and caused no visible clinical changes at all, which potentially could be a considerable advantage to its clinical use. Although at this early stage, we have not carried out experimental tests of genotoxicity, we have carried out a theoretical prediction exercise. Osiris Property Explorer [57] is a tool widely used to evaluate the drug-score of potential compounds to meet potential candidate drug requirements. The toxicity risk assessment is predicted through a process based on comparison with a precomputed set of structural fragments which come from the toxic group from the RTECS database (Registry of Toxic Effects of Chemical Substances), maintained by US National Institute for Occupational Safety and Health (NIOSH). Toxicity data scores generated include primary irritation; mutagenic effects; reproductive and tumorigenic effects; acute toxicity and multiple dose toxicity. When EPI and its stereoisomers were evaluated through this tool (http://www.organic-chemistry.org/prog/peo/ accessed on February 01th 2015), no toxicity risks were found. Similarly, we also compared the structural features of EPI to a published of Pan Assay INterference compounds or PAINs groups, promiscuous, and assay-interfering activities, and found no match [58]. Nevertheless, such prediction tools are no substitute for experimental testing and, considerable further testing is required in the future regarding the safety of this new alkaloid, specifically genotoxicity and reproductive toxicity data will be needed.

The results of pKa shown that the EPI can be used safely at physiological pH without any loss of activity or appearance of toxic derivatives.

The results of in vitro assays with many drugs do not correspond to what is observed in vivo. In fact, what works well in vitro may not work in vivo and vice versa, as shown by Katz et al., 2013 [59]. Our article clearly shows—in *S*. *mansoni*, considerable differences in a drug’s efficacy in vitro and in vivo. EPI is not very potent in vitro—this is a fact. But the response that we measured in vivo (40 mg/kg) was reasonable (n = 10), this number of animals is appropriate to the experiment according to the literature.

An important practical aspect to be considered is that EPI can be extracted and purified from the waste of the production of pilocarpine from jaborandi leaves (*Pilocarpus microphylus*), so that it is expected that total production costs compared to other active pharmaceutical ingredients would be very low.

### Conclusions

In conclusion EPI significantly reduced parasite worm burden and the alkaloid showed no measurable toxicity in the model tested, at concentrations superior to effective treatment concentrations. At this time, EPI is a promising candidate drug. More studies are needed to evaluate the therapeutical effectiveness of EPI, and to evaluate the efficacy of this drug against different stages of the life cycle as well as other species of *Schistosoma*. In the future, pharmacokinetic studies to explore the fate of the drug in the body are required. Additionally, the detailed mechanism of action of EPI in schistosomes remains to be investigated with other preclinical tests.
